# PLANT-BASED DIETS AND CARDIOVASCULAR DISEASE RISK FACTORS IN OLDER ADULTS: AN INTEGRATIVE LITERATURE REVIEW

**DOI:** 10.1093/geroni/igae098.3297

**Published:** 2024-12-31

**Authors:** Tricia VanCleef

**Affiliations:** Fran and Earl Ziegler College of Nursing, University of Oklahoma Health Sciences, Oklahoma City, Oklahoma, United States

## Abstract

Cardiovascular disease (CVD) is the leading cause of mortality globally, disproportionately impacting older adults. Plant-based diets (PBDs) have emerged as a dietary strategy to mitigate CVD risk, characterized by foods derived from plants and minimal to no animal-derived foods. This integrative review assesses the effectiveness of PBDs for improving CVD risk factors in older adults. A search was conducted in CINAHL, PubMed, and Medline databases for studies of PBDs and CVD risk factors in individuals aged 65 years and older. Quality assessment was performed using the Mixed Methods Appraisal Tool (MMAT). 17 publications were analyzed, 7 experimental and 10 non-experimental. All studies showed at least one positive association between PBDs and cardiovascular health in older adults. Findings highlighted significant associations between PBD adherence and reductions in key CVD risk factors such as low-density lipoprotein cholesterol (LDL-C), blood pressure, and body mass index (BMI), alongside potential benefits in reducing coronary artery calcium and enhancing endothelial function and serum isoflavone concentrations. There is evidence for the potential of PBDs as an effective dietary strategy to prevent the onset and progression of CVD in older adults and reduce the risk of CVD mortality. This review indicates the need for further research, specifically larger randomized control trials focused on older adults to strengthen the evidence base and to guide healthcare recommendations. PBDs should be considered as a part of holistic CVD prevention and management strategies for older adults, encouraging dietary patterns that are rich in fruits, vegetables, legumes, beans, nuts, seeds, and whole grains.

